# Separation of Bacteria, Protozoa and Carbon Nanotubes by Density Gradient Centrifugation

**DOI:** 10.3390/nano6100181

**Published:** 2016-10-12

**Authors:** Monika Mortimer, Elijah J. Petersen, Bruce A. Buchholz, Patricia A. Holden

**Affiliations:** 1 Bren School of Environmental Science and Management, Earth Research Institute and University of California Center for the Environmental Implications of Nanotechnology (UC CEIN), University of California, Santa Barbara, CA 93106, USA; mmortimer@bren.ucsb.edu; 2 Laboratory of Environmental Toxicology, National Institute of Chemical Physics and Biophysics, Akadeemia tee 23, Tallinn 12618, Estonia; 3 Biosystems and Biomaterials Division, Material Measurement Laboratory, National Institute of Standards and Technology, Gaithersburg, MD 20899, USA; elijah.petersen@nist.gov; 4 Center for Accelerator Mass Spectrometry, Lawrence Livermore National Laboratory, Livermore, CA 94550, USA; buchholz2@llnl.gov

**Keywords:** *Pseudomonas aeruginosa*, *Tetrahymena thermophila*, carbon-14, sucrose, iodixanol, bioaccumulation, bioconcentration, Stokes’ Law

## Abstract

Sustainable production and use of carbon nanotube (CNT)-enabled materials require efficient assessment of CNT environmental hazards, including the potential for CNT bioaccumulation and biomagnification in environmental receptors. Microbes, as abundant organisms responsible for nutrient cycling in soil and water, are important ecological receptors for studying the effects of CNTs. Quantification of CNT association with microbial cells requires efficient separation of CNT-associated cells from individually dispersed CNTs and CNT agglomerates. Here, we designed, optimized, and demonstrated procedures for separating bacteria (*Pseudomonas aeruginosa*) from unbound multiwall carbon nanotubes (MWCNTs) and MWCNT agglomerates using sucrose density gradient centrifugation. We demonstrate separation of protozoa (*Tetrahymena thermophila*) from MWCNTs, bacterial agglomerates, and protozoan fecal pellets by centrifugation in an iodixanol solution. The presence of MWCNTs in the density gradients after centrifugation was determined by quantification of ^14^C-labeled MWCNTs; the recovery of microbes from the density gradient media was confirmed by optical microscopy. Protozoan intracellular contents of MWCNTs and of bacteria were also unaffected by the designed separation process. The optimized methods contribute to improved efficiency and accuracy in quantifying MWCNT association with bacteria and MWCNT accumulation in protozoan cells, thus supporting improved assessment of CNT bioaccumulation.

## 1. Introduction

Continuing nanomaterial development and production for various applications in consumer products [1] and agriculture [2] have increased the need for rapid and reliable hazard assessment of engineered nanomaterials. An important part of hazard assessment is determining the bioaccumulation and biomagnification potential of engineered nanomaterials, including carbon nanotubes (CNTs). This requires quantification of intracellular nanomaterials or nanomaterials associated with organisms, after effective removal of the unbound or loosely bound nanomaterials from the organisms. Bacteria, and eukaryotic unicellular organisms, such as protozoa and algae, provide good systems for studying environmental hazards of engineered nanomaterials [3,4,5,6,7,8]. However, microbes also pose challenges in nanomaterial accumulation studies because cells need to be separated from nanomaterials, which in aqueous media form agglomerates in a similar size range as the cells.

Different approaches have been used to separate unicellular organisms from unbound nanomaterials, for example, repetitive washing and centrifugation to isolate the alga *Chlorella vulgaris* from single-wall carbon nanotubes (SWCNTs) and graphene oxide [9], and filtration to remove CdSe quantum dots from bacteria such as *Pseudomonas aeruginosa* [10]. Recently, a differential centrifugation method was validated for separation of bacteria-associated iron oxide nanoparticles (NPs) from free NPs [11]. The latter method efficiently removed free, colloidally stable, NPs from NP-coated *Staphylococcus aureus*. However, differential centrifugation would not be as efficient in separating colloidally less stable nanomaterials, such as CNTs, from CNT-associated bacteria, because CNTs can form bacteria-sized agglomerates [12]. In addition, differential centrifugation may be inefficient in separating motile organisms like ciliates from free CNTs. Still, the mobility of the ciliated protozoan *Tetrahymena thermophila* has been utilized in isolating such unicellular organisms from pellets that also contain SWCNTs and bacteria, by pelleting the sample by centrifugation and then allowing ciliates to swim out of the pellet into the supernatant wherein they are collected [13]. Although elegant, this approach would not be suitable for isolating ciliates prior to quantifying the intracellular CNT content because, during the time required for the protozoa to emerge from the pellet, the ciliates would start to clear their food vacuole contents, thus introducing artifacts in measuring nanomaterial uptake.

Density gradient centrifugation can be used to separate cells or particles based on their size and mass (rate-zonal centrifugation) or density (isopycnic centrifugation). As such, density gradient centrifugation has been widely employed for separating microorganisms. For example, a sucrose density gradient method was found to be effective in size-based separation of aquatic microbes [14] and for isolating ciliate species of *Uronema* from bacteria [15]. Sucrose gradient centrifugation has also been successfully used in size-sorting of nanomaterials [16,17], including multiwall carbon nanotubes (MWCNTs) [18]. Although employed for separation of either microorganisms or CNTs, sucrose density gradients have not been previously used for separating microorganisms from free MWCNTs. Because they are biocompatible and non-ionic, sucrose solutions are advantageous in isolating live bacteria for further use in in vitro assays or for trophic transfer experiments. Sucrose is easily washed from the cells, and bacteria tolerate the high viscosity and osmolarity of the concentrated solution during short centrifugation periods. Eukaryotic microbes, however, have been reported to become destroyed in concentrated sucrose gradients and thus require either fixation before centrifugation in sucrose or use of a different density gradient medium for separation of live cells. For example, polysucrose has been used to separate *T. thermophila* from virus particles [19], and colloidal silica suspensions have been used to separate the green alga *Desmodesmus subspicatus* from unbound MWCNTs [20]. However, neither polysucrose nor colloidal silica solutions are ideal for density gradient separation of eukaryotic cells, especially ciliates, because of the high viscosity and hyperosmotic properties of the former [14], and because the latter is comprised of particles that are ingestible by phagocytosing ciliates [21,22].

Here, we report density gradient centrifugation methods optimized for separation of (i) the bacteria *P. aeruginosa* from free MWCNTs in a sucrose density gradient and (ii) *T. thermophila* from bacteria, MWCNTs and fecal pellets, using nonionic isosmotic iodixanol solutions. Bacteria were separated from MWCNTs in a sucrose density gradient by rate-zonal centrifugation while the isolation of protozoa in iodixanol solutions employed elements of both rate-zonal and isopycnic centrifugations. Specifically, in rate-zonal centrifugation, where particles are separated based on their size and sedimentation rate, the density of the particles and cells must exceed the density of the gradient medium, so that objects migrate into the medium during centrifugation [23]. Isopycnic centrifugation on the other hand separates particles solely on the basis of their buoyant densities in the gradient media, and the particles remain in their isopycnic zone independent of centrifugation time. Here, we employed relatively low centrifugation speeds for a short time that is optimal for isolating live cells, while the gradient media were chosen to separate the system components based on size when separating bacteria from MWCNTs, and either size or buoyant density in isolation of protozoa. These approaches allowed for fast and convenient isolation of live microorganisms for further use in in vitro assays or for quantification of intracellular or strongly cell-associated MWCNTs. The detection and quantification of MWCNTs in the density gradients, as well as quantifying MWCNTs associated with isolated microbes, were achieved by the use of ^14^C-labelled MWCNTs (^14^C-MWCNTs). Optical microscopy was used to assess the purity of the bacterial and protozoan cells recovered from density gradients. Protozoan cell recovery rates after the centrifugation steps were assessed for four different growth phases: lag, exponential, late exponential and stationary. The separation procedure was carried out both with MWCNT-exposed microorganisms and control cultures without MWCNTs. The method described herein in detail was successfully applied in our recent study that assessed bioaccumulation of MWCNTs in *Tetrahymena thermophila* [24].

## 2. Results and Discussion

### 2.1. MWCNT Characteristics and Associations with Bacteria

MWCNTs used in the study were generally shorter than 500 nm, with an average diameter of 36.5 nm ± 12.7 nm (*n* = 80, uncertainty values indicate standard deviation throughout the paper), and surface area of 111 m^2^/g, as reported previously [25]. The non-carbon content of the MWCNTs was 5.8% ± 2% (average of three measurements ± standard deviation) and the nickel catalyst residue was determined to be 2.6 mg/g MWCNTs. The specific activity of the ^14^C-MWCNTs was 555 kBq/g (0.015 mCurie/g) [24].

The MWCNTs were coated with oxygen-containing functional groups as a result of acid functionalization, as determined by X-ray photoelectron spectroscopy (XPS) performed previously [26], affording stability in the aqueous suspensions and thus increasing the probability of exposure to the microorganisms. The interactions between bacteria and MWCNTs were promoted by suspending the MWCNTs in Nanopure water by probe-sonication which apparently changed the physical properties of the MWCNTs compared to the MWCNT powder as seen in the environmental scanning electron microscopy (ESEM) images (Figure S1). MWCNTs appeared less tangled after probe-sonication which is expected based on the known effects of high-energy probe-sonication processes on MWCNTs [27]. Based on XPS performed previously [26], 6-h probe-sonication also increased the surface oxygen content of MWCNTs from 7.4% to 8.6%, further contributing to the stability of the stock suspensions. In this study, most 88% ± 2% of the MWCNTs remained stably dispersed by the 4th day after sonication and the suspension remained stable for at least 6 months. The hydrodynamic diameter of MWCNT agglomerates in the bacterial growth medium was approximately 320 nm (Table 1). It should be noted, however, that the hydrodynamic diameter measurements are calculated using assumptions for spherical particles, thus are not directly applicable for fibrous or rod-shaped nanomaterials and should be used as general guide to the dimensions of MWCNTs and their agglomerates in the suspension.

**Figure 1:**
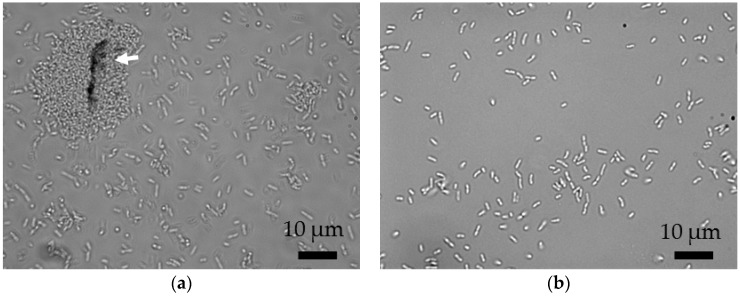
Representative phase contrast images of *P. aeruginosa* incubated with 5 mg/L MWCNTs for 1 h: (**a**) pelleted bacteria after differential centrifugation, white arrow points to MWCNT agglomerate; and (**b**) bacteria after density gradient centrifugation in sucrose.

During the co-incubation of bacteria and MWCNTs, individual MWCNT agglomerates could have readily interacted with bacterial cells that have average dimensions of 1.4 µm × 0.6 µm. Because MWCNTs could form larger agglomerates, and due to the observed agglomerates of bacteria and MWCNTs by optical microscopy (Figure 1a), repetitive washing and differential centrifugation were insufficient for separation of bacteria from MWCNT agglomerates (data not shown). Therefore, and to exclude any possible contamination of the bacterial fraction by the unbound MWCNTs, density gradient centrifugation was performed following differential centrifugation.

### 2.2. Separation of MWCNT-Associated Bacteria from Unbound MWCNTs

Bacteria were separated from unbound MWCNTs using a two-step centrifugation: (i) differential centrifugation; and (ii) density gradient centrifugation in sucrose solutions. In the differential centrifugation step, where particles are separated based on their size and density, free MWCNTs were estimated to sediment along with bacteria, mainly because the calculated minimum diameter of MWCNTs that would sediment based on Stokes’ Law [28] was smaller (Table 2) than the measured hydrodynamic diameter of MWCNTs in the bacterial growth medium (Table 1). Therefore, subsequent density gradient centrifugation was deemed necessary for complete separation of MWCNT-encrusted bacteria from unbound MWCNTs. According to the published literature, gram-negative rod-shaped bacteria localize in an approximately 55% to 60% sucrose layer during isopycnic centrifugation, meaning that their buoyant density is equal to the density of sucrose in this concentration range [23,29]. Thus, the gradient of 50% and 60% (*w*/*v*) sucrose was chosen for separation of bacteria and MWCNTs by rate-zonal density gradient centrifugation.

**Scheme 1:**
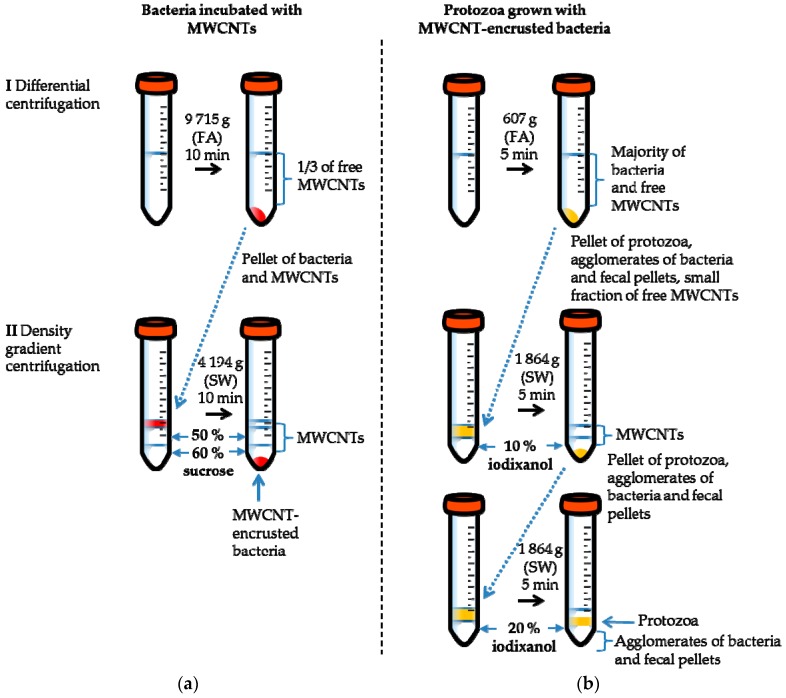
Schematic illustration of observed separation results for bacteria and protozoa after either bacterial exposure to MWCNTs, or protozoan exposure to bacterial prey with their cell-associated MWCNTs: (**a**) separation of MWCNT-associated bacteria (shown in red) from MWCNTs; and (**b**) separation of MWCNTs, bacteria and fecal pellets from protozoa (shown in yellow) that were grown with MWCNT-encrusted bacteria; free MWCNTs are included as a component of the system to account for possible “shedding” of MWCNTs from bacteria before or after the uptake into protozoan food vacuoles. Blue lines denote liquid levels in the tubes and interfaces of different liquids. FA—fixed angle rotor, SW—swinging bucket rotor (Table S2).

The calculated minimum diameter of bacteria required for pelleting in 50% sucrose was slightly larger (Table 2) than the assumed diameter of bacteria (Table 3) and thus, theoretically, the bacteria were not expected to sediment in 50% sucrose. However, considering that the diameter was calculated assuming a sphere of equivalent volume to bacteria and that the value was very similar to the calculated diameter (Table 2), bacterial sedimentation in the sucrose gradient was tested experimentally. Differently from theoretical predictions (Table 2), bacteria pelleted in the sucrose gradient (Scheme 1a). In a previous study, the formation of bacterial pellets in 100% (*w*/*v*) sucrose was reported when a similar centrifugal force and time were used as in the current study [30]. The increased removal of bacteria in sucrose, relative to what was expected, could have been caused by interactions of sucrose molecules with the bacterial cell envelope by coating the cells or diffusing into the periplasm through the pores in the outer membrane [31]. As a result of these interactions the cell diameter would slightly increase, which would explain the sedimentation of bacteria in 50% and 60% sucrose. However, despite the possible interactions of sucrose with bacteria, the bacteria recovered from the pellet beneath the sucrose density gradient 75% ± 14% of initial bacterial number before centrifugation) were viable and axenic as confirmed by observing their growth on Luria-Bertani (LB) agar plates compared to bacteria that were not exposed to sucrose or centrifuged (data not shown).

The localization of MWCNTs in the centrifugation media was tested experimentally, employing ^14^C-MWCNT suspensions in bacteria-free spent culture broth, i.e., a cell-free medium containing bacterial extracellular polymeric substances (EPS) and other metabolites. In this experiment, two thirds (66% ± 9%) of the total ^14^C-MWCNTs pelleted in the differential centrifugation step and thus would be transferred with pelleted bacteria into the sucrose density gradient (Scheme 1a). After density gradient centrifugation, nearly one third (27% ± 4%) of the transferred ^14^C label remained in the 0.5 mL aqueous sample above the sucrose layers; 54% ± 9% was in the upper 1 mL of the 50% sucrose layer, and 19% ± 4% was in the bottom 1 mL of the 50% sucrose layer (Scheme 1a). The ^14^C mass concentration was below the detection limit (0.3 µg MWCNTs/mL) in the 60% sucrose layer, indicating that unassociated MWCNTs were unlikely to pellet with bacteria through the sucrose density gradient and thus would be retained in the 50% sucrose layer. The sedimentation of MWCNTs in the sucrose density gradient, differently from theoretical estimations that were based on the calculated minimum diameter of MWCNTs that would sediment, likely indicated slight agglomeration of MWCNTs when suspended in the medium containing bacterial EPS. The cell-free experiment with ^14^C-MWCNTs might have not mimicked MWCNT sizes and agglomerate formation in the presence of bacteria with absolute accuracy because of high adsorption affinity of MWCNTs to bacteria, which would make MWCNT homoagglomerate formation unlikely. However, the experiments with ^14^C-MWCNT suspensions indicated that possible MWCNT agglomerates would be separated from bacteria by the sucrose density gradient centrifugation (Scheme 1a). Following the density gradient separation procedure for bacteria, large agglomerates of MWCNTs, which would have been apparent in the microscopy images, were not observed in the recovered pellet, thus providing additional evidence that the MWCNT agglomerates were either separated from bacteria (Figure 1b) or were not present at the beginning of the density gradient centrifugation.

The repeatability of the separation protocol was confirmed by conducting the experiment thrice, each performed in triplicate; the coefficient of variation (CV) for percent cell recovery in sucrose was 20% and for the MWCNT mass associated with bacteria, 10%. Additionally, it was confirmed with ^14^C-MWCNT-spiked sucrose solutions that sucrose did not interfere with ^14^C quantification (data not shown). In addition, the bacterial cell recovery in the sucrose gradient was the same for MWCNT-encrusted bacteria and control bacteria (without MWCNTs, data not shown) indicating that MWCNT adsorption to bacteria did not increase the size or mass of bacteria to an extent that would have changed their sedimentation rate in the sucrose density gradient during centrifugation. Consequently, the density of bacteria without MWCNTs is reported in Table S1 and used in calculations of the minimum diameters of cells that would sediment (Table 2).

Slightly less than half (44% ± 5%) of the nominal added MWCNTs were associated with the bacterial cells after the separation procedure as confirmed by ^14^C measurements. In general, a high adsorption affinity of MWCNT clusters to bacteria has been reported in the literature in the contexts of potential applications such as removal of MWCNTs from effluents using bacteria [32] and employing MWCNTs for capturing and removing bacteria [33]. However, some MWCNTs may have been unaccounted for, possibly due to adsorption of MWCNTs (dispersed or homoagglomerated) to bacterial agglomerates attached to the glass Erlenmeyer flask walls, such that not all MWCNTs were available to adsorb to bacterial cells in the suspension. To assess possible loss to glassware adsorption, ^14^C-MWCNTs were quantified in the total bacterial suspension without any separation procedure. In the latter, recovery of ^14^C-MWCNTs relative to the controls (uninoculated media) that were spiked with ^14^C-MWCNTs and immediately quantified for ^14^C using liquid scintillation counting (LSC) was 64% ± 12% of the nominal added MWCNTs, thus indicating that a large loss to glassware adsorption was possible and could explain the low recovery of MWCNTs on bacteria. However, the recovery could also vary with varying the proportions of MWCNT to bacterial cell concentrations, although this was not further studied.

### 2.3. Protozoan Growth by Predation of Bacteria with and without MWCNTs

**Figure 2:**
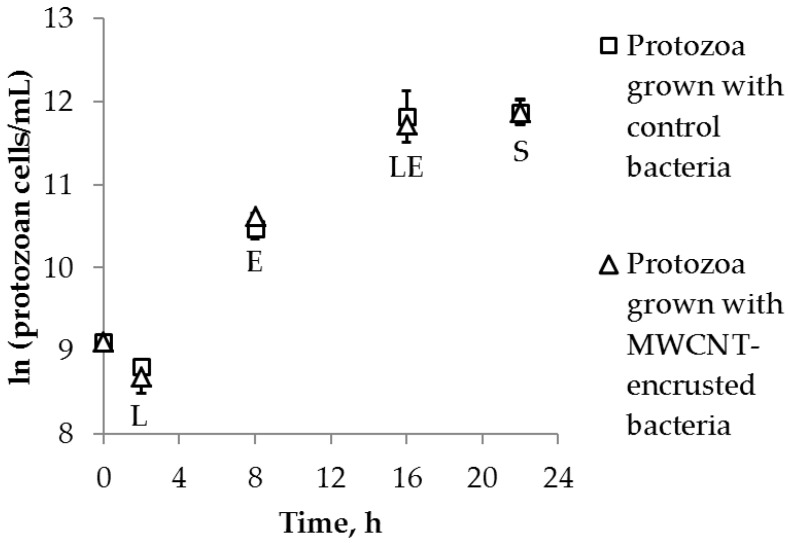
Natural log-transformed *T. thermophila* cell counts per milliliter over 22 h growth with control or MWCNT-treated *P. aeruginosa* as a food source. Data points are the average of three replicates, and error bars indicate standard deviation values. Letters designate growth phases: L—lag phase, E—exponential phase, LE—late exponential phase, and S—stationary phase.

**Figure 3:**
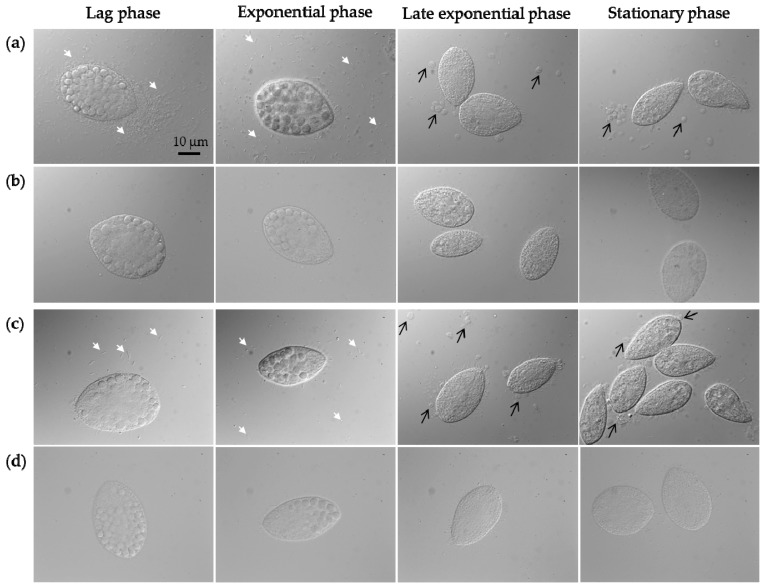
Nomarski images of *T. thermophila* grown with MWCNT-encrusted bacteria (**a**,**b**) and control bacteria (**c**,**d**), taken before (**a**,**c**) and after (**b**,**d**) separation steps (differential centrifugation and density gradient centrifugations in iodixanol) at different growth phases. The round shapes inside protozoan cells are food vacuoles filled with bacteria. White arrows indicate bacteria provided as food source and black arrows show protozoan fecal pellets, i.e., secreted food vacuoles containing digested or partly digested bacteria.

The ciliated protozoa *T. thermophila* were grown with *P. aeruginosa* that had previously been incubated with MWCNTs and isolated by sucrose density gradient centrifugation as described above. The bacteria and protozoa were suspended in a non-nutrient mineral medium (Dryl’s) that did not support microbial growth, such that the bacteria were the only food source for protozoa while the bacteria did not multiply. The ciliate growth was not affected by MWCNTs: the specific growth rates (0.21 h^−1^ ± 0.02 h^−1^ and 0.18 h^−1^ ± 0.02 h^−1^) and maximum yields (1.5 × 10^5^ ± 2 × 10^4^ cells/mL and1.5 × 10^5^ ± 3 × 10^4^ cells/mL) were similar in the cultures grazing on control bacteria and on MWCNT-encrusted bacteria, respectively (Figure 2). The ciliates grazed on bacteria by phagocytosis through the protozoan oral apparatus (cytostome), forming food vacuoles (phagosomes) inside the cells to digest the bacteria. Undigested matter was ejected as fecal pellets through the cytoproct at the opposite end of the protozoan cell. The intracellular food vacuoles packed with bacteria were visible in all four protozoan growth stages, with the smallest number of vacuoles in the stationary phase cultures (Figure 3). In general, the internalization of MWCNTs was not readily observable in the images due to bacteria occupying most of the space in the protozoan phagosomes. However, the uptake of MWCNTs by protozoa via grazing on MWCNT-encrusted bacteria was confirmed by ^14^C-MWCNT quantification as described in Materials and Methods and in Section 2.2. *T. thermophila* cultures sampled in the lag and exponential phases contained considerable amounts of single bacterial cells, compared to later growth phase cultures, which were rich in fecal pellets consisting of digested and undigested bacterial agglomerates (Figure 3a,c). The MWCNTs supplied to protozoa were associated with bacteria as confirmed by quantification of ^14^C-MWCNTs, but the presence of free MWCNTs in the media, by release from the bacteria before or after the uptake into *T. thermophila* food vacuoles, could not be excluded and thus was considered as one of the components of the system.

### 2.4. Separation of Protozoa from Bacteria, Fecal Pellets, and MWCNTs

Similarly to the method for bacterial isolation from MWCNTs in a sucrose gradient, the protozoan samples were first concentrated by differential centrifugation. As protozoa require lower centrifugation speeds and shorter times for pelleting than bacteria, this step would separate most of the freely suspended bacteria and MWCNTs from protozoa as estimated theoretically (Table 3). However, as differential centrifugation is known to result in incomplete separation of the sample components, and since bacterial agglomerates and fecal pellets of protozoa were estimated to pellet with the protozoa during the differential centrifugation step, subsequent density gradient centrifugation steps were required for isolation of protozoa.

Although sucrose was suitable for separating bacteria from MWCNTs, and despite a previous report of using a sucrose density gradient for separating a ciliate species from bacteria [15], herein centrifugation in sucrose was found to deform and shrink live *T. thermophila* cells, as observed by optical microscopy, likely due to the hyperosmolality of the concentrated sucrose solutions (not shown). This is consistent with a previous report where sucrose could be used as a separation medium for protozoa only after fixing the cells with formaldehyde [14]. To achieve isolation of live ciliate cells, we used an iodixanol density gradient medium, which is a non-toxic, nonionic isosmotic aqueous solution. The concentrations of iodixanol solutions (10% and 20%, *w*/*v*) used for isolation of protozoa were chosen based on reported buoyant density of *Toxoplasma gondii*, a parasitic protozoa, in Nycodenz^®^ solution (an iodinated density gradient medium with similar properties to iodixanol) [34]. In the reported protocol, a gradient of 10% to 30% Nycodenz^®^ was used for isolating protozoa by isopycnic density gradient centrifugation and the protozoan cells were harvested in the density zone of 1090 kg/m^3^ to 1110 kg/m^3^ that corresponds to 20% iodixanol (Table S1).

In the current study, centrifugations in 10% and 20% iodixanol were conducted in two consecutive steps instead of a gradient of a 10% solution overlain on 20% iodixanol. This was because the preliminary experiments indicated that the ciliated protozoa did not form a distinct band in the gradient, resulting in low recovery rates of protozoa (data not shown). The use of a 10% iodixanol medium was expected to allow for separation of protozoa from MWCNTs and bacteria based on different sizes, i.e., following rate-zonal centrifugation principles (Table 3). Subsequent centrifugation in 20% iodixanol was estimated to isolate protozoa into a band above the 20% iodixanol layer, while bacterial agglomerates and fecal pellets of protozoa were expected to pellet based on their higher buoyant density in iodixanol compared to that of protozoa, and thus would be separated by isopycnic centrifugation (Table S1 and Table 3).

Protozoa, grown with bacteria as a sole food source, were sampled at four different time points, representing different growth phases of protozoan populations (Figure 2), to test if the growth phase and varying number of bacteria and fecal pellets would influence the recovery rate of protozoa in the density gradient centrifugation. As mentioned previously, the bacteria did not multiply in the protozoan media, so the number of bacteria decreased and the number of fecal pellets increased over time as the protozoa grazed on bacteria and ejected the digested and partly digested bacteria as fecal pellets (Figure 3a,c). Although MWCNTs were supplied to protozoa via MWCNT-encrusted bacteria, the presence of free MWCNTs in the media, by release from the bacteria before or after the uptake into protozoan food vacuoles, could not be excluded and thus was considered as one of the components of the system (Table 3 and Scheme 1b).

^14^C-MWCNT suspensions in protozoa-free Dryl’s medium that contained protozoan EPS were employed to determine experimentally the localization of MWCNTs in the centrifugation media. Based on these experiments, most of the MWCNTs and their agglomerates (80% ± 3%) remained in the supernatant during the differential centrifugation step, and 20% ± 6% was transferred into the next centrifugation step with 10% iodixanol. After density gradient centrifugation, all of the ^14^C activity (92% ± 24% of the transferred ^14^C-MWCNT mass) was recovered in the top 10% iodixanol layer (2.5 mL), confirming that any free MWCNTs would be separated from protozoa that would pellet in this step (Scheme 1b).

**Figure 4:**
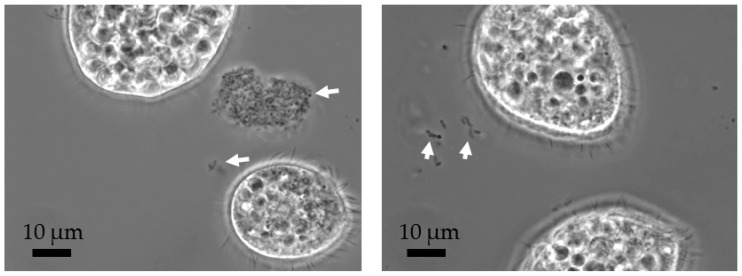
Representative phase contrast images of the pelleted fraction after centrifugation of *T. thermophila* suspensions through 10% iodixanol. The two images serve as replicates and are both shown since bacterial agglomerates (**left** image) and free bacterial cells (**right** image) were each observed. White arrows indicate bacterial agglomerates or cells that pelleted with the protozoan cells.

**Figure 5:**
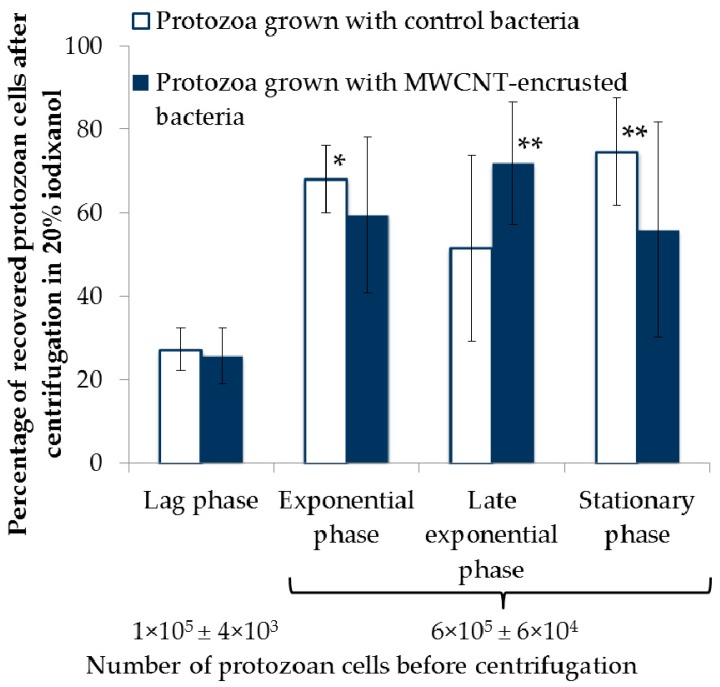
Protozoan cell recovery percentages after density gradient centrifugation steps in iodixanol when sampled at four different protozoan growth phases. Protozoa were grown with control (untreated, white bars) or MWCNT-treated bacteria (blue bars). Data bars represent the mean values of three measurements and error bars are standard deviations. The values of “Number of protozoan cells before centrifugation” denote the total protozoan cell number in the concentrated samples after differential centrifugation but before density gradient centrifugation in iodixanol. Asterisks indicate significant difference from the recovery percentage at lag phase, both in control and MWCNT-amended exposure, using Tukey’s multiple comparisons test across all eight conditions, *p* ≤ 0.05 (**) and *p* ≤ 0.1 (*). There was no significant difference between the percentages of recovered protozoan cells grown with control bacteria (white bars) versus protozoan cells grown with MWCNT-encrusted bacteria (blue bars) within each growth phase.

Bacterial agglomerates and fecal pellets, composed of intact and partly digested bacteria, were estimated to pellet along with protozoa in 10% iodixanol (Table 3) by assuming that the buoyant density of bacteria in iodixanol was 1130 kg/m^3^ (Table S1). The pellet that formed after centrifugation in 10% iodixanol did contain protozoa, bacterial agglomerates and fecal pellets, which was apparent from phase contrast images (Figure 4). Then, in the next density centrifugation step, the purity of protozoan cells collected from the top of the 20% iodixanol layer was confirmed by optical microscopy and indicated a similar purity at each sampling point at different growth phases, regardless of whether the protozoa were grown with control bacteria or MWCNT-encrusted bacteria (Figure 3b,d). Similarly, there was no statistically significant difference between the numbers of protozoan cells recovered from the 20% iodixanol when protozoan cultures were grown with control or MWCNT-encrusted bacteria (Figure 5). However, the protozoan recovery rate was lower (around 30%) in the samples harvested during the lag phase than in the later time points (ranging from 50% to 75%). The samples were concentrated by differential centrifugation with an aim to achieve a similar number of protozoa to be transferred to the 10% iodixanol step at each sampling point, and thus required harvesting larger volumes of protozoan cultures at earlier growth phases when the cell concentration was lower. However, the lag phase cultures had very low cell counts (Figure 2) and it was not feasible to harvest large enough volumes to reach the cell concentrations equal to later growth phase samples. Thus, the lower recovery rate of protozoa at the first sampling point (Figure 5) likely indicated the dependence of the recovery on the cell concentration before density gradient centrifugation rather than the growth phase, although the cell concentration was not systematically varied to confirm this hypothesis. The percent recovery of the protozoan cells was repeatable in two independent experiments, each performed in triplicate, with the average CV values of data from both experiments being 22%, 43%, 25% and 41% at lag, exponential, late exponential and stationary phase, respectively.

**Figure 6:**
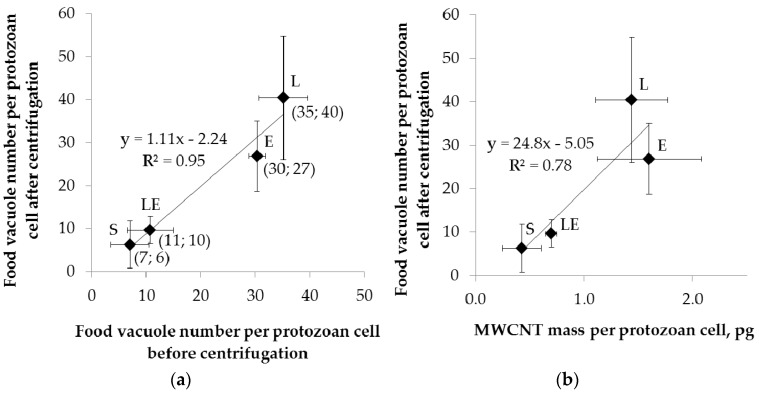
(**a**) Comparison of food vacuole numbers in protozoan cells before and after differential and density gradient centrifugations; and (**b**) correlation between the food vacuole numbers and MWCNT mass per protozoan cell when sampled at four different growth phases (indicated by the letters: L—lag phase, E—exponential phase, LE—late exponential phase and S—stationary phase). Numbers at each data point in panel (**a**) indicate mean food vacuole numbers before and after centrifugation (*x*; *y*), which were not statistically different based on the two-sample *t*-test (*p* ≤ 0.05) for each of the growth phases. Food vacuole numbers per cell are the mean values of 5 to 11 individual cells. MWCNT masses are based on the mean values of three LSC measurements of ^14^C associated with radiolabeled MWCNTs. Error bars indicate standard deviations.

Because ciliates are motile and feed by phagocytosis, the duration of the separation procedure should be kept as short as possible to reduce the likelihood of the ciliates ingesting the density gradient medium. Iodixanol is reported to be non-toxic to cells [35], and thus would not be harmful to ciliates if ingested. However, there was a concern that phagocytosis of iodixanol could possibly accelerate the ejection of the contents of food vacuoles filled with bacteria and MWCNTs. To confirm that the intracellular content of the ciliates was not affected by the separation procedure, the food vacuole numbers in the protozoa were quantified in the Nomarski microscopy images taken before and after the separation by differential centrifugation followed by density gradient centrifugations (Figure 3 and Figure 6a). The food vacuole numbers were greatest in the cells harvested at lag phase, i.e., when the ratio of bacteria to protozoa was the highest; the food vacuole numbers decreased over time, with the lowest food vacuole numbers counted in stationary phase cultures. Nonetheless, there was no statistically significant difference in the food vacuole numbers per protozoan cell before and after the overall separation procedure at any of the four sampling points as indicated by the slope of the regression line being near one and the associated high R^2^ value (Figure 6a). After separating protozoan cells by density gradient centrifugation in iodixanol, the ^14^C label (associated with the trophically transferred MWCNT mass that was internalized by protozoa) was quantified by LSC, as reported elsewhere [24]. Because the vacuole numbers and the measured MWCNT mass per protozoan cell (again, by LSC of ^14^C label) showed a generally similar trend with higher vacuole numbers corresponding to higher MWCNT mass per cell (Figure 6b) employing the food vacuole number as an indicator of the relative amount of internalized MWCNTs was justified.

## 3. Materials and Methods

### 3.1. MWCNT Synthesis and Characterization

MWCNTs and ^14^C-MWCNTs were synthesized using a chemical vapor deposition technique as described previously [36,37]. Briefly, methane and a mixture of ^14^C-labeled methane and regular methane were used as feedstock gases to produce MWCNTs and ^14^C-MWCNTs, respectively. The resulting nanotubes were purified using concentrated hydrochloric acid, and treated with a 3:1 (volume fraction) mixture of sulfuric to nitric acid to render them more hydrophilic [37]. Physico-chemical characterization was performed with unlabeled MWCNTs synthesized by the same method as ^14^C-MWCNTs. The surface area of the acid-treated nanotubes was measured by the standard Brunauer–Emmett–Teller (BET) method; the lengths and diameters of MWCNTs were measured in scanning electron microscopy (SEM) images, as reported previously [25]. Non-carbon content of MWCNTs was recorded as percent residual weight of the sample at 900 °C during thermogravimetric analysis (TGA; Thermogravimetric Analyzer/sDTA 851e, Mettler Toledo, Columbus, OH, USA) performed according to the ISO/TS 11308:2011 standard [38]. Briefly, approximately 10 mg of MWCNTs were weighed on a microbalance into an alumina sample pan with an outer diameter of 6 mm and height of 4.5 mm (AdValue Technology, Tucson, AZ, USA). Samples were heated from 25 °C to 900 °C at a rate of 5 °C/min in 50 mL/min air flow. TGA scans were conducted for three separate samples. An empty sample pan was run as a blank and subtracted from sample results to correct for buoyancy effects. The catalyst residue content of the MWCNTs was measured by inductively coupled plasma optical emission spectroscopy (ICP-OES; iCAP™ 6300 ICP-OES Analyzer, Thermo Scientific, Waltham, MA, USA). The ICP-OES samples were prepared by digesting the remaining MWCNT residue after the TGA analysis in aqua regia (concentrated plasma-pure HNO_3_ and HCl, 1:3, *v/v*) at 100 °C for 1 h, then diluting with Nanopure water to a final acid concentration of 2%.

### 3.2. MWCNT Suspension Preparation and Characterization

Stock suspensions of MWCNTs and ^14^C-MWCNTs at 200 mg/L in Nanopure water were prepared by ultrasonication at 40% amplitude for 1 h, pulsing for 30 s on and 10 s off (Cole-Parmer 750 Watt Ultrasonic Homogenizer (Vernon Hills, IL, USA) with a 13-mm diameter probe and replaceable tip). The output power, measured as described previously [39], was 27 W. The MWCNT and ^14^C-MWCNT dispersion stability was checked by the optical density measurements at 600 nm and the ^14^C specific activity measurements using LSC as described in Section 3.10, respectively. The stock dispersions were kept at room temperature in the dark until the experiments. The z-average diameters of 10 mg/L MWCNTs in Nanopure water and in test media were measured using a Zetasizer Nano ZS-90 (Malvern Instruments, Westborough, MA, USA). MWCNTs were imaged by environmental scanning electron microscopy (ESEM) as a dry powder and as an air-dried stock suspension on a double sided adhesive copper conductive tape mounted on an aluminum stub (Ted Pella, Redding, CA, USA), using an FEI XL30 (Hillsboro, OR, USA) with a field emission gun (FEG; Philips Electron Optics, Eindhoven, the Netherlands) operated at a 15 kV accelerating voltage with a gaseous secondary electron detector in wet mode (3 torr).

### 3.3. P. aeruginosa Culturing and Incubation with MWCNTs

*P. aeruginosa* PG201, maintained and cultured as previously described [10,40,41,42], was grown in Erlenmeyer flasks containing 50 mL of growth medium (half-strength 21C [43]: 0.5 g/L NH_4_Cl, 1.725 g/L Na_2_HPO_4_·7H_2_O, 1.38 g/L KH_2_PO_4_ and 1% *v*/*v* Hutner’s mineral solution [44]). Glucose at 3.4 g/L was added as a carbon source. The cultures were incubated at 30 °C, 250 rpm (26.2 rad/s) until the optical density at 600 nm (OD_600_) reached 0.7 (ca. 18 h). The ^14^C-MWCNT stock dispersion (mixed with 2× concentrated growth medium at a ratio of 1:1, *v*/*v*) was added to bacterial culture yielding a final nominal ^14^C-MWCNT concentration of 1 mg/L. Similar exposures with unlabeled MWCNTs and cells without added MWCNTs were included as controls and for microscopic cell counts and imaging. Bacteria were incubated with or without MWCNTs at 30 °C, 250 rpm (26.2 rad/s) for 1 h.

### 3.4. Differential Centrifugation and Sucrose Density Gradient Centrifugation of Bacteria

Sucrose was dissolved in Dryl’s medium (2 mmol/L sodium citrate, 2 mmol/L NaH_2_PO_4_·H_2_O, 1 mmol/L Na_2_HPO_4_, 1.5 mmol/L CaCl_2_, pH 7.4) to achieve 50% and 60% (*w*/*v*) sucrose solutions. Sucrose solutions were sterilized by filtration through syringe filters (pore size 0.2 μm, Thermo Scientific Nalgene Syringe Filter, 25 mm surfactant free cellulose acetate membrane) and carefully pipetted into sterile conical polypropylene 15 mL centrifuge tubes (Corning Incorporated, Corning, NY, USA) by adding a 2 mL layer of 50% sucrose over a 2 mL layer of 60% sucrose. Late exponential phase bacterial culture (10 mL), incubated with or without MWCNTs, was concentrated by differential centrifugation at 9715 *g* (Sorvall RC-5B Plus, fixed angle rotor SLA-1500, Table S2) for 10 min, 10 °C, and resuspended in 0.5 mL of Dryl’s medium, vortexed for 60 s, then pipetted carefully over the sucrose gradient for density gradient centrifugation. The tube contents were centrifuged at 4194 *g* for 10 min, 10 °C, using a swinging-bucket rotor (Sorvall SH-3000, Table S2). The supernatant (top and sucrose fractions, 4.5 mL total) was removed and discarded. The pellet was washed by vortexing (1 min) in 10 mL of Dryl’s medium, followed by centrifugation at 9715 *g* for 10 min, 10 °C. The washing step was repeated and the pellet was suspended in Dryl’s medium by vortexing. The suspended bacteria were used for protozoan exposures to MWCNTs (Section 3.6) and microscopic cell counting and imaging (Section 3.11). For the cells that had been exposed to ^14^C-MWCNTs, ^14^C associated with the cells was quantified by LSC as described in Section 3.10.

### 3.5. Theoretical Estimations of Centrifugal Separation Parameters

In the differential centrifugation steps, centrifugation times and forces routinely used to pellet bacteria and protozoa were employed (Table 2 and Table 3) [10,42]. The relative centrifugal forces needed to sediment bacteria and protozoa—without consideration for MWCNTs—in the respective density gradient media were also based on published literature [30,34]. Centrifugation times (*t*) of density gradient centrifugations were adjusted based on Stokes’ Law (Equation (1)) to ensure separation of the system components [28]:

(1) $$t=\frac{18\mu \;ln\left(\frac{R\max}{R\min}\right)}{\omega^{2}d^{2}\left(\rho_{p}‒\rho_{m}\right)}$$

where μ is absolute viscosity of the medium (kg/(m·s)), *R*_max_ is the radial distance of the bottom of the centrifuge tube from the center of rotation (corresponds to the maximum radius of rotor and the position of pelleted cells after centrifugation, m), *R*_min_ is the radial distance of the liquid level in the centrifuge tube from the center of rotation (corresponds to the position of the cells before centrifugation, m), ω is the angular velocity (rad/s), ρ*_p_* and ρ*_m_* are the densities of the cells and the medium, respectively (kg/m^3^), and *d* is the cell diameter (m). The diameters of bacteria and protozoa were assumed to correspond to diameters of spheres of equivalent volume to bacteria (0.37 µm^3^) and protozoa (7042 µm^3^, Table 1). The viscosities and densities of the media, as well as the densities of the cells used in the calculations are listed in Table S1. As per Table S1, buoyant densities of cells suspended in either sucrose or iodixanol are reported, because these density gradient media result in slightly altered densities of cells (as measured by others). These calculated centrifugation times (Equation (1)) were then employed (Scheme 1).

However, although these times were employed, the likelihood of effective separations was assessed theoretically, again using Stokes’ Law. For assessing probable separation of bacteria from MWCNTs and protozoa from MWCNTs, fecal pellets and bacterial agglomerates, the actual particle diameters (Table 1) were compared to the theoretical minimum diameters (*d*, in meters or m) calculated as those that would sediment according to Stokes’ Law [45] (from Equation (2), Table 2 and Table 3). If the minimum particle size that would theoretically sediment was less than the actual size, the actual particle was predicted to sediment. When the actual particle size was less than that predicted to sediment, then it was assumed that sedimentation would not occur:

(2) $$d=\sqrt{\frac{18\mu \;ln\left(\frac{R\max}{R\min}\right)}{\omega^{2}\left(\rho_{p}‒\rho_{m}\right)t}}$$

It should be mentioned that the centrifugations were performed at 10 °C but the theoretical estimations based on Stokes’ Law used viscosity and density values of the media at 20 °C because of the unavailability of the data at 10 °C for all the media (Table S1). However, when the minimum diameters of the system components that would pellet were calculated using the viscosity and density values of water at 10 °C and then at 20 °C, the difference in calculated diameters did not change the sedimentation rate estimations (data not shown).

### 3.6. Culturing of T. thermophila and Exposure to MWCNTs

The protozoan *T. thermophila* SB210E, maintained and cultured as described previously [10,42], was inoculated into 10 mL of rich medium (1% SSP: 1% proteose peptone, 0.1% yeast extract, 0.2% dextrose, 0.003% iron ethylenediaminetetraacetic acid (Fe-EDTA)) in standard (10 cm by 15 mm) sterile plastic Petri dishes and grown in a humidity chamber without shaking for 17 h, 30 °C. Late exponential-phase cultures were harvested by differential centrifugation at 607 *g* (fixed angle rotor SLA-1500, Table S2) for 10 min, 10 °C. The cell pellet was washed once with Dryl’s medium and resuspended to a concentration of ca. 650,000 cells/mL. The cells in Dryl’s medium were pipetted into Petri dishes (10 mL) and starved in humidity chambers (17 h, 30 °C).

For protozoan exposure to MWCNTs, *P. aeruginosa* with associated MWCNTs (unlabeled or ^14^C-labeled), were recovered from the sucrose density gradient then washed and resuspended in Dryl’s medium (10 mL; Section 3.4) to an average cell concentration of 1 × 10^8^ cells/mL. This suspension was pipetted into Petri dishes, to which starved *T. thermophila* cells were added to achieve an initial protozoan cell concentration of ca. 10^4^ cells/mL. Separate cultures of *T. thermophila* that were fed control *P. aeruginosa* (not exposed to MWCNTs) were included as controls. *T. thermophila* was cultured in the dark in a humidity chamber (30 °C) without agitation. The cultures were harvested at 2 h, 8 h, 16 h and 22 h of growth which corresponded to the lag phase, exponential, late exponential and stationary phases, respectively.

### 3.7. Differential Centrifugation of Protozoan Exposures and Density Gradient Centrifugation in Iodixanol Solutions

Iodixanol, obtained as an OptiPrep™ (Axis-Shield, Oslo, Norway) density gradient medium which is a sterile and endotoxin free (≤0.13 endotoxin units/mL) solution of 60% (*w/v*) iodixanol in water with a density of 1.32 g/mL, was used to separate *T. thermophila* from bacteria and fecal pellets. After growth with *P. aeruginosa* as prey, *T. thermophila* was harvested by low-speed differential centrifugation at 607 *g* for 5 min at 10 °C. Most (9 mL) of the supernatant was discarded and the bottom 1 mL was retained to resuspend the pellet which contained concentrated protozoa, fecal pellets, and bacterial agglomerates. The concentrated suspension was gently pipetted over 2 mL of 10% iodixanol in water in a 15 mL clear polypropylene centrifuge tube (Corning, Corning, NY, USA) and centrifuged at 1864 *g* for 5 min, 10 °C, in a swinging-bucket rotor (Sorvall SH-3000, Table S2). The supernatant (top layer and iodixanol solution) was removed from the pellet; the pellet was resuspended in 500 µL of Dryl’s medium and gently pipetted on top of 2 mL 20% iodixanol in a 15 mL centrifuge tube. The centrifugation was repeated at 1864 *g* for 5 min, 10 °C. The top 2 mL which contained the protozoan cells based on their lower density as compared to fecal pellets or bacterial agglomerates (Table S1), was pipetted into a clean 15 mL tube; Dryl’s medium was added to increase the volume to 10 mL, and the contents were mixed by rotating the tube gently then centrifuged at 607 *g* for 5 min, 10 °C, to wash the cells. The pellet was suspended in Dryl’s medium and used for microscopic cell counting and imaging as described in Section 3.11. The cells exposed to ^14^C-MWCNTs via bacterial prey were digested, and ^14^C was quantified by LSC as described in Section 3.10.

### 3.8. Determining ^14^C-MWCNT Localization in the Sucrose Gradient

Because the use of sucrose gradients for separating MWCNTs from bacterial cells is unproven, we needed to perform cell free density gradient separation experiments to determine which fraction of the sucrose solutions would likely be associated with MWCNTs upon sedimentation. We performed these trial MWCNT-sucrose localization experiments by first suspending MWCNTs in cell-free spent culture broth, since extracellular substances in the cultures could have influenced MWCNT agglomeration before density gradient centrifugation. *P. aeruginosa* cells were grown without MWCNTs as described in Section 3.3 to late exponential phase and harvested by differential centrifugation (9715 *g*, 10 min). The supernatant was collected and centrifuged again (9715 *g*, 10 min) to remove all the bacteria. ^14^C-MWCNTs were suspended in the resulting cell-free supernatant to achieve a ^14^C-MWCNT concentration of 1 mg/L. For total activity measurements, 2.5 mL of the resulting suspension was mixed with 2.5 mL of liquid scintillation cocktail for measurement of the ^14^C activity by LSC. The next steps with the 1 mg/L MWCNT suspension were performed following the separation protocol for bacteria and MWCNTs, except without bacteria: 10 mL of 1 mg/L MWCNT suspension was pipetted into a 15 mL centrifuge tube and centrifuged (9715 *g*, 10 min); the supernatant was discarded except for 200 µL which was estimated to be approximately the same volume of medium remaining with the bacterial pellet following differential centrifugation. Dryl’s medium (500 µL) was added to the remaining 200 µL, suspended by pipetting, and this suspension was then pipetted carefully over the sucrose gradient (50% sucrose over 60% sucrose) prepared as described in Section 3.4. Sucrose gradients were centrifuged (4194 *g*, 10 min, 10 °C) in a swinging-bucket rotor (Sorvall SH-3000, Table S2). The top 0.7 mL aqueous layer and 1 mL aliquots of the sucrose fractions were pipetted into LSC vials, Dryl’s medium was added to each sample to bring the volume to 2.5 mL, mixed with 2.5 mL liquid scintillation cocktail, and ^14^C was quantified by LSC as described in Section 3.10.

### 3.9. Determining ^14^C-MWCNT Localization in the Iodixanol Media

Because the use of iodixanol for separating MWCNTs from protozoan cells is unproven, we needed to perform cell free density gradient separation experiments to determine which fraction of the iodixanol solutions would likely be associated with MWCNTs upon sedimentation. We performed these trial MWCNT-iodixanol localization experiments by first suspending MWCNTs at 1 mg/L in cell-free Dryl’s medium containing *T. thermophila* extracellular substances, since extracellular substances in the cultures could have influenced MWCNT agglomeration before density gradient centrifugation. To prepare cell-free medium *T. thermophila* was cultured in SSP medium then starved overnight in Dryl’s medium as described in Section 3.6. The starved *T. thermophila* cells were centrifuged at 607 *g* for 10 min; the supernatant was collected and centrifuged again (607 *g*, 10 min). The resulting cell-free supernatant was used to suspend ^14^C-MWCNTs. Total activity of the 1 mg/L suspensions was measured by LSC as described in Section 3.10. To experimentally determine the sedimentation of ^14^C-MWCNTs in the iodixanol media used to isolate protozoa, the steps of the separation protocol were performed with the cell-free ^14^C-MWCNT suspensions. Specifically, 10 mL of the 1 mg/L MWCNT suspension was pipetted into each of nine 15 mL centrifuge tubes and centrifuged at 607 *g* for 5 min. For three tubes, ^14^C activity was measured in the fractions (from top to bottom: 3 × 2.5 mL, 1 × 2 mL and 1 × 0.5 mL) of the differentially centrifuged suspensions. For the remaining tubes, the bottom 0.5 mL which was estimated to be approximately the same volume of medium remaining with the protozoan pellet following differential centrifugation, was mixed with 0.5 mL Dryl’s medium, and the resulting 1 mL was transferred over the 10% iodixanol solution. After centrifugation at 1864 *g* for 5 min, three tubes were used to quantify the ^14^C activity in the top 2.5 mL and bottom 0.5 mL (location of protozoa in the separation experiments) fractions of the 10% iodixanol gradients, as described in Section 3.10. From the remaining three tubes, the top 2.5 mL were discarded and the bottom 0.5 mL transferred over the 20% iodixanol. After centrifugation at 1864 *g* for 5 min, the ^14^C activity was quantified by LSC for the top 2.5 mL (location of protozoa in the separation experiments) and bottom 0.5 mL fractions.

### 3.10. Liquid Scintillation Counting (LSC)

^14^C-MWCNTs were quantified by measuring the activity of ^14^C using LSC. Bacterial and protozoan cell pellets from density gradient centrifugations were suspended in 2.5 mL of 0.1% sodium dodecyl sulfate (SDS)/0.1 mol/L NaOH, vortexed and heated at 55 °C for 45 min to digest the cells. To quantify total suspended ^14^C-MWCNTs in the bacterial cultures, 1.5 mL of bacterial culture was mixed with 1 mL 0.1% SDS/0.1 mol/L NaOH to digest bacteria as in the pellets. The digested samples were mixed with 2.5 mL of liquid scintillation cocktail (UltimaGold XR, Perkin Elmer, Groningen, The Netherlands) and kept in the dark for 1 h before quantifying ^14^C by LSC (LS 6500, Beckman Coulter Inc., Fullerton, CA, USA). Quenching of ^14^C by bacterial and protozoan samples was between 5% and 10% which was measured by spiking unamended samples (cell pellets or cell suspensions) with a known mass of ^14^C-MWCNTs, then quantifying the radiolabel as a fraction of the spiked amount, using scintillation counting.

In the cell-free experiments, the fractions of centrifugation gradients were directly mixed with 2.5 mL liquid scintillation cocktail. If less than 2.5 mL was sampled from the gradient, the volume was brought to 2.5 mL by adding Dryl’s medium prior to mixing with 2.5 mL liquid scintillation cocktail. Samples mixed with the cocktail were kept in the dark for 1 h before quantifying ^14^C by LSC.

### 3.11. Protozoan and Bacterial Cell Counting, Optical Microscopy, and Food Vacuole Counting

Bacteria and protozoa were fixed with 2.5% glutaraldehyde after sampling for microscopic cell counting and imaging, and kept at 4 °C until analysis. Fixed *P. aeruginosa* cells were stained with SYBR Gold (Life Technologies, Carlsbad, CA, USA) and counted as described previously [46]. Briefly, glutaraldehyde-fixed cell suspensions were diluted 100× with phosphate buffered saline (PBS, pH 7.4); 150-µL aliquots were combined with 2.55 mL of PBS and 300 µL of a 500× dilution of SYBR Gold in 15 mL foil-wrapped conical tubes (Corning, Corning, NY, USA). The suspension was incubated at room temperature in the dark for 20 min and then filtered through an Anodisc filter (25-mm diameter, 0.2-μm pore size; Whatman), then mounted on a standard glass slide, and sealed with a coverslip. Three replicate slides were prepared for each sample. Cells were counted using an epifluorescent microscope (Nikon E800, 1000× magnification, Melville, NY, USA) with an ocular micrometer grid. Cells were counted in at least 10 randomly chosen microscope fields. A total of at least 200 bacteria were counted per slide. The average numbers of bacteria per milliliter were calculated from the results of 3 replicate slides with a CV of less than 10%.

*T. thermophila* cells were counted in a hemocytometer using bright field microscopy (Olympus CX41, 100× magnification, Center Valley, PA, USA). The cell density of the fixed sample was adjusted to allow for counting approximately 20–50 cells total in four corner sections, each 3 × 3 mm, of a 9 × 9 mm Neubauer grid. Each sample was loaded in the chamber and counted at least in duplicate. The CV ranged between 2% and 20%.

For microscopic imaging, 5 μL of the cell suspensions were pipetted onto a glass slide, mixed with 5 μL of Mowiol^®^ 4-88 (Sigma-Aldrich, St. Louis, MO, USA), covered with a glass coverslip, allowed to harden at room temperature for 24 h, and stored at 4 °C. An Olympus BX51 upright microscope with differential interference contrast optics (Nomarski microscopy, Center Valley, PA, USA) was used for imaging, with a Retiga 2000R QImaging Camera (Surrey, BC, Canada) with Q-Capture Pro 7 software (Surrey, BC, Canada) for image capturing. Several images at different focal planes were acquired from each *T. thermophila* cell to visualize intracellular food vacuoles. A Nikon E800 upright microscope with a camera and RS Image software (Roper Scientific, Trenton, NJ, USA) were used for phase contrast imaging and image capturing, respectively.

The food vacuoles in *T. thermophila* cells were manually counted from the Nomarski images using the multi-point tool in ImageJ [47]. Five to eleven cells were analyzed per time point for each treatment.

### 3.12. Statistical Analysis

Statistically significant differences of the means were determined using either a two-tailed Student’s *t*-test (Microsoft Excel, Microsoft Corporation, Redmond, WA, USA) or one-way analysis of variance (ANOVA) and post hoc Tukey’s multiple comparisons test (R, version 2.15.1). The values reported are the means of at least 3 measurements ± standard deviation.

## 4. Conclusions

In conclusion, methodologies were developed for physicochemically separating bacteria from MWCNTs, and for separating protozoan cells from bacterial prey and protozoan fecal pellets. Such methods are needed to allow for precisely quantifying the distribution of MWCNTs in bioaccumulation and trophic transfer studies using microorganisms, which are at the base of food webs. The quantification herein of MWCNTs was afforded by incorporating ^14^C as a label into the MWCNT structure, and using liquid scintillation counting of ^14^C as the MWCNT tracer. Alternatively, when unlabeled CNTs are employed in such studies, other methods with similar limit of detection as LSC could be used for quantification of CNTs, e.g., inductively coupled plasma-mass spectrometry (ICP-MS), single-particle ICP-MS or inductively coupled plasma optical emission spectroscopy (ICP-OES) using metal catalyst impurities as proxies to quantify CNTs, Raman spectroscopy, and, in the case of SWCNTs, near-infrared fluorescence spectroscopy [48]. A sucrose gradient centrifugation procedure was well suited for separating bacteria from MWCNTs and their agglomerates. However, sucrose was not deemed suitable for separating protozoan predators from MWCNT-encrusted or control bacterial cells, and from MWCNT agglomerates and fecal pellets, because of the known deleterious effects of sucrose on protozoa. Instead, protozoa were effectively separated from other trophic transfer experimental components using a novel density gradient centrifugation method based on iodixanol solutions. A theoretical approach, applying Stokes’ Law, was used to optimize centrifugal times in density gradient centrifugations and to assess the likelihood of effective separations by calculating the theoretical minimum diameters of the particles that would sediment in both differential and density gradient centrifugation. It was demonstrated that, in case of density gradient media such as sucrose that interact with cell surfaces or permeate the cell membrane, possible discrepancies between the theoretical and experimental results should be considered. Separation based on size and mass (rate-zonal centrifugation) was most appropriate in separating bacteria from MWCNTs, and a combination of size- and buoyant density-based separation was applied when isolating protozoa. Short centrifugation times required for separating the cells from other components of the system minimized the contact of live cells with the density gradient media and thus did not affect cell viability or MWCNT-association with the cells. It should be noted that, in the current study, the density gradient separation procedures were optimized for isolation of bacteria purified from free MWCNTs (in sucrose gradient) and protozoa from purified from bacteria, MWCNT agglomerates, and fecal pellets (in iodixanol gradient) to allow for quantification of the cell-associated MWCNTs. If bacteria-free fractions of MWCNTs in sucrose gradient or protozoa-free fractions of bacteria or fecal pellets in iodixanol gradient are desired, different optimal conditions should be employed. In addition, other medium chemistries and exposure components might dictate different optimal solutions, concentrations, centrifugation speeds, and times. However, the overall concept and application of theory and empiricism are highly transferable.

## Figures and Tables

**Table 1:** Hydrodynamic diameters of MWCNTs in test media, diameters of bacteria in half-strength 21C medium and diameters of protozoa and other components of the system in Dryl’s medium.

Component of the System	Diameter, m
MWCNTs in Dryl’s medium	1.7 × 10^−7^ ± 2 × 10^−9^ (0.21) ^a^
MWCNTs in bacterial growth medium (half-strength 21C)	3.2 × 10^−7^ ± 1 × 10^−8^ (0.24) ^a^
Bacteria (*P. aeruginosa*)	8.9 × 10^−7^ ^b^
Protozoa (*T. thermophila*)	2.4 × 10^−5^ ^b^
Fecal pellets of protozoa	4.0 × 10^−6^ ^c^
Bacterial agglomerates	<1 × 10^−5^ and >8.9 × 10^−7^ ^d^

^a^ The MWCNTs in the test media result from diluting a probe-sonicated stock dispersion to a final concentration of 10 mg/L in the medium; z-average diameters are the mean values of 3 replicate measurements ± standard deviation (Polydispersity Index); ^b^ diameters of a sphere of equivalent volume to bacteria (0.37 µm^3^) or protozoa (7042 µm^3^), respectively; ^c^ diameters as measured in Nomarski images (Figure 3); ^d^ size range according to measurements in Nomarski images (Figure 3).

**Table 2:** Parameters employed in separating bacteria from MWCNTs and estimated minimum diameters of MWCNTs and bacteria that would pellet (Column 9) at the relative centrifugal forces and centrifugation time used in the experiments.

1. Centrifugation Step	2. Medium	3. Medium Volume, mL	4. Path Length of the Particles in the Medium, m	5. Centrifugation Time, s	6. Rotor Maximum Radius, m	7. Rotor Minimum Radius, m ^a^	8. Relative Centrifugal Force (RCF) ^b^	9. Calculated Particle Diameter (m, Equation (2)) with Expected Sedimentation (Y/N)	
								MWCNTs	Bacteria
Differential centrifugation	Half-strength 21C	10	0.08	600	0.1359	0.0559	9715	2.8 × 10^−7^ (Y) ^c^	7.3 × 10^−7^ (Y) ^d^
Density gradient centrifugation	Dryl’s	0.5	0.003					2.7 × 10^−7^ (N) ^e^	7.1 × 10^−7^ (Y) ^d^
	50% sucrose	2	0.014					8.4 × 10^−7^ (N) ^e^	**1.6 × 10^−6^ (N)** ^f^
	60% sucrose	2	0.027					1.4 × 10^−6^ (N) ^e^	**4.3 × 10^−6^ (N)** ^f^
		4.5	0.044	600	0.1854	0.1414	4194		

^a^ Calculated by subtracting column 4 from column 6; ^b^ calculated as shown in Table S2; Y—calculated diameter based on Stokes’ Law was smaller than the assumed diameter of the particle or cell (Table 1), and thus the system component would theoretically pellet; N—calculated diameter was larger than the assumed diameter of particle or cell (Table 1), thus the system component would theoretically not pellet; ^c^ assuming diameter as measured in half-strength 21C; ^d^ assuming a buoyant density of bacteria in water of 1070 kg/m^3^ (Table S1); ^e^ assuming diameter as measured in Dryl’s medium (Table 1); ^f^ assuming a buoyant density of bacteria in sucrose of 1260 kg/m^3^ (Table S1); bold values—not consistent with experimental results, likely because the cell size or density were changed by the interactions between sucrose solution and bacteria.

**Table 3:** Parameters employed in separating protozoa from bacteria, fecal pellets and MWCNTs, and estimated minimum diameters of the system components that would pellet (Column 9) at the relative centrifugal forces and centrifugation time used in the experiments.

1. Centrifugation Step	2. Medium	3. Medium Volume, mL	4. Path Length of the Particles in the Medium, m	5. Centrifugation Time, s	6. Rotor Maximum Radius, m	7. Rotor Minimum Radius, m ^a^	8. Relative Centrifugal Force (RCF) ^b^	9. Calculated Particle Diameter (m, Equation (2)) with Expected Sedimentation (Y/N)				
								MWCNTs ^c^	Bacteria ^d^	Bacterial Agglomerates ^e^	Protozoa ^d^	Fecal Pellets of Protozoa ^d^
Differential centrifugation	Dryl’s	10	0.08	300	0.1359	0.0559	607	1.6 × 10^−6^ (N) ^f^	4.1 × 10^−6^ (N) ^h^	4.1 × 10^−6^ (Y) ^h^	4.7 × 10^−6^ (Y) ^j^	4.1 × 10^−6^ (Y) ^h^
Density gradient centrifugation I	Dryl’s	1	0.006					4.9 × 10^−7^ (N) ^f^	1.3 × 10^−6^ (N) ^h^	1.3 × 10^−6^ (Y) ^h^	1.5 × 10^−6^ (Y) ^j^	1.3 × 10^−6^ (Y) ^h^
	10% iodixanol	2	0.027					7.0 × 10^−7^ (N) ^f^	1.7 × 10^−6^ (N) ^i^	1.6 × 10^−6^ (Y) ^i^	2.0 × 10^−6^ (Y) ^k^	1.6 × 10^−6^ (Y) ^i^
		3	0.033	300	0.1854	0.1524	1864					
Density gradient centrifugation II	Dryl’s	0.5	0.003					NA	NA	1.2 × 10^−6^ (Y) ^h^	1.4 × 10^−6^ (Y) ^j^	1.2 × 10^−6^ (Y) ^h^
	20% iodixanol	2	0.027					NA	NA	3.1 × 10^−6^ (Y) ^i^	NC	3.1 × 10^−6^ (Y) ^i^
		2.5	0.03	300	0.1854	0.1554	1864					

^a^ Calculated by subtracting column 4 from column 6; ^b^ calculated as shown in Table S2; ^c^ free MWCNTs were included as theoretical components of the system, to account for possible release of MWCNTs from the bacteria before or after the uptake into protozoan food vacuoles; ^d^ assumed diameters as shown in Table 1; ^e^ assumed diameter < 1 × 10^−5^ (Table 1); Y—calculated diameter based on Stokes’ Law was smaller than the assumed diameter of the particle or cell (Table 1), and thus the system component would theoretically pellet; N—calculated diameter was larger than the assumed diameter of particle or cell (Table 1), thus the system component would theoretically not pellet; ^f^ assuming a diameter as measured in Dryl’s medium (Table 1); ^h^ assuming a buoyant density of bacteria in water of 1070 kg/m^3^ (Table S1); ^i^ assuming a buoyant density of bacteria in iodixanol of 1130 kg/m^3^ (Table S1); ^j^ assuming a buoyant density of protozoa in water of 1054 kg/m^3^ (Table S1); ^k^ assuming a buoyant density of protozoa in iodixanol of 1107 kg/m^3^ (Table S1); NA—not applicable, the component was not transferred from density gradient centrifugation step I to step II as confirmed experimentally; NC—not calculated because density of 20% iodixanol was equal to the assumed buoyant density of protozoa.

## Supplementary Materials

The following are available online at www.mdpi.com/2079-4991/6/10/181/s1.

## Abbreviations

The following abbreviations are used in this manuscript:

^14^C-MWCNT	^14^C-labeled multiwall carbon nanotubes
BET	Brunauer–Emmett–Teller
CNT	carbon nanotubes
CV	coefficient of variation
LSC	liquid scintillation counting
MWCNT	multiwall carbon nanotubes
NP	nanoparticles
SWCNT	single-wall carbon nanotubes
TGA	thermogravimetric analysis
