# Supplementary material for: Separation of Bacteria, Protozoa and Carbon Nanotubes by Density Gradient Centrifugation
Source: Nanomaterials (Basel). 2016 Oct 12;6(10):181. doi: 10.3390/nano6100181 (PMC5132190; doi:10.3390/nano6100181)
Supplement: Supplementary file 1 [file nanomaterials-06-00181-s001.pdf]

# Supplementary Materials: Separation of Bacteria, Protozoa and Carbon Nanotubes by Density Gradient Centrifugation

**Monika Mortimer, Elijah J. Petersen, Bruce A. Buchholz and Patricia A. Holden**

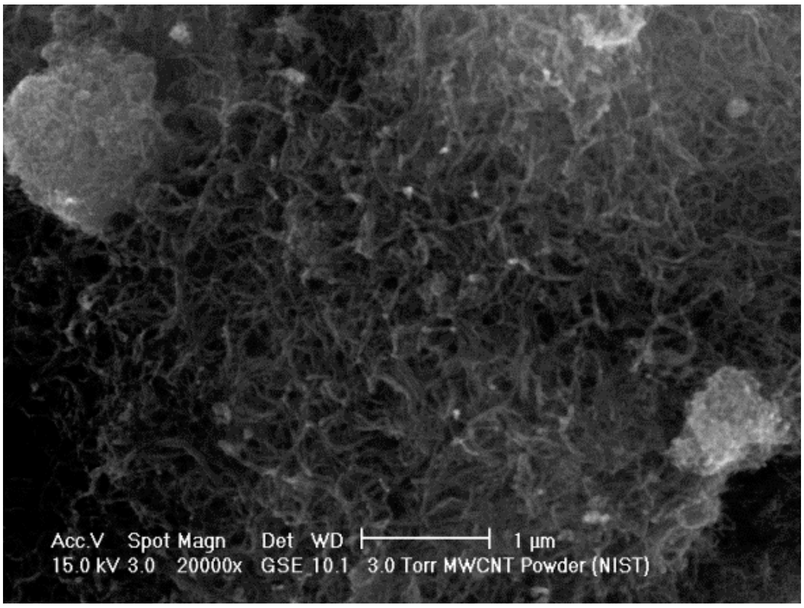


(**a**)

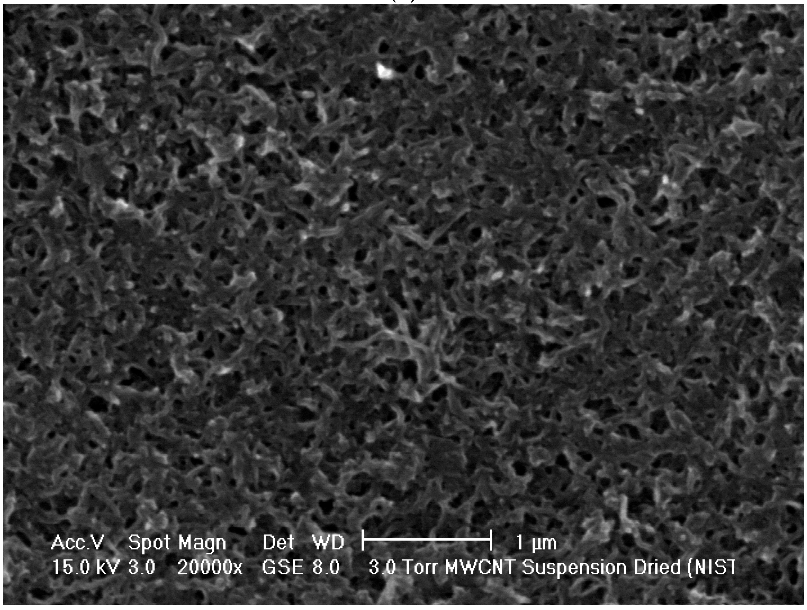


(**b**)

**Figure S1.** Environmental scanning electron microscopy images of multiwall carbon nanotubes (MWCNTs). (**a**) MWCNT powder; (**b**) probe-sonicated MWCNT suspension, air-dried. Both samples were imaged at a 15-kV accelerating voltage with a gaseous secondary electron detector in wet mode (3 torr); scale bar is 1 µm. MWCNTs appeared less tangled after probe-sonication in Nanopure water, reflected by the overall lower distribution density and less notable bending of MWCNTs (**b**) which likely facilitated their association with bacterial cells.

**Table S1.** Parameters used in calculating cell and particle diameters in application of Stokes' Law for estimating separation of the system components in various density gradient compositions.

| Parameter | Value | Units |
|---|---|---|
| Viscosity of water at 20 °C | 0.001002 [a] | kg/(m·s) |
| Density of water at 20 °C | 998.2 [a] | kg/m$^3$ |
| Viscosity of 50% (*w*/*v*) sucrose at 20 °C | 0.0062 [b] | kg/(m·s) |
| Density of 50% (*w*/*v*) sucrose at 20 °C | 1176 [b] | kg/m$^3$ |
| Viscosity of 60% (*w*/*v*) sucrose at 20 °C | 0.0155 [b] | kg/(m·s) |
| Density of 60% (*w*/*v*) sucrose at 20 °C | 1230 [b] | kg/m$^3$ |
| Viscosity of 10% (*w*/*v*) iodixanol at 20 °C | 0.00185 [c] | kg/(m·s) |
| Density of 10% (*w*/*v*) iodixanol at 20 °C | 1053 [c] | kg/m$^3$ |
| Viscosity of 20% (*w*/*v*) iodixanol at 20 °C | 0.002 [c] | kg/(m·s) |
| Density of 20% (*w*/*v*) iodixanol at 20 °C | 1107 [c] | kg/m$^3$ |
| Density of MWCNTs | 1500 [d] | kg/m$^3$ |
| Buoyant density of bacteria in water | 1070 [e] | kg/m$^3$ |
| Buoyant density of bacteria in sucrose | 1260 [f] | kg/m$^3$ |
| Buoyant density of bacteria in iodixanol | 1130 [g] | kg/m$^3$ |
| Buoyant density of protozoa in water | 1054 [h] | kg/m$^3$ |
| Buoyant density of protozoa in iodixanol | 1107 [i] | kg/m$^3$ |
| Density of fecal pellets of protozoa, containing bacteria, in water | 1070 [j] | kg/m$^3$ |
| Density of fecal pellets of protozoa, containing bacteria, in iodixanol | 1130 [j] | kg/m$^3$ |

[a] Crittenden et al., 2012 [1]; [b] Asadi 2005 [2]; [c] Axis-Shield Applications and Products 2015 [3]; [d] Laurent et al., 2010 [4]; [e] Inoue et al., 2007 [5]; [f] Eroglu and Melis, 2008 [6]; [g] Henriquez et al., 2003 [7]; [h] Harvey et al., 1997 [8]; [i] assumed to be equal to the density of 20% iodixanol based on Axis-Shield Application Sheet C51 [9]; [j] assumed to be the same as density of bacteria in the respective media.

**Table S2.** Rotor parameters, centrifugal velocities and respective relative centrifugal forces used in the study.

| Rotor Type and Model | Rotor Maximum Radius, cm | Revolutions per Minute, rpm | Relative Centrifugal Force (RCF) [a] |
|---|---|---|---|
| Sorvall fixed angle rotor SLA-1500 | 13.59 | 2000 | 607 |
| | | 8000 | 9715 |
| Sorvall swinging bucket rotor SH-3000 | 18.54 | 3000 | 1864 |
| | | 4500 | 4194 |

[a] Relative centrifugal force (RCF) was calculated as follows: $RCF = (R_{max}/100) \times (\omega^2)/g$ where $R_{max}$ is rotor maximum radius in centimeters which is divided by 100 to convert to units of meters; $\omega$ is the rotor angular velocity (rad/s) which is calculated by multiplying the revolutions per minute (rpm) by $2\pi/60$; $g$ is the constant of gravitational acceleration (9.8 m/s$^2$).

## References


1. Crittenden, J.C.; Trussell, R.R.; Hand, D.W.; Howe, K.J.; Tchobanoglous, G. *MWH's Water Treatment: Principles and Design*, 3rd ed.; John Wiley & Sons, Inc.: New York, NY, USA, 2012.
2. Asadi, M. *Beet-Sugar Handbook*; John Wiley & Sons, Inc.: New York, NY, USA, 2005.
3. Axis-Shield, Density Gradient Media. Applications and Products. Available online: http://www.axis-shield-density-gradient-media.com/Iodinated%20density%20gradient%20media.pdf (accessed on 22 May 2015).
4. Laurent, C.; Flahaut, E.; Peigney, A. The weight and density of carbon nanotubes versus the number of walls and diameter. *Carbon* **2010**, *48*, 2994–2996.
5. Inoue, K.; Nishimura, M.; Nayak, B.B.; Kogure, K. Separation of marine bacteria according to buoyant density by use of the density-dependent cell sorting method. *Appl. Environ. Microbiol.* **2007**, *73*, 1049–1053.
6. Eroglu, E.; Melis, A. "Density" equilibrium method for the quantitative and rapid in situ determination of lipid, hydrocarbon, or biopolymer content in microorganisms. *Biotechnol. Bioeng.* **2009**, *102*, 1406–1415.

7. Henriquez, V.; Rojas, M.V.; Marshall, S.H. An alternative efficient procedure for purification of the obligate intracellular fish bacterial pathogen *Piscifickettsia salmonis*. *Appl. Environ. Microbiol.* **2003**, *69*, 6268–6271.
8. Harvey, R.W.; Metge, D.W.; Kinner, N.; Mayberry, N. Physiological considerations in applying laboratory-determined buoyant densities to predictions of bacterial and protozoan transport in groundwater: Results of in-situ and laboratory pests. *Environ. Sci. Technol.* **1997**, *31*, 289–295.
9. *Application Sheet C51. Purification of Toxoplasma Gondii from Cell Cultures*; Axis-Shield: Dundee, Scotland, UK, 2013.
